# The ‘placement’ of people with profound impairments across the lifespan: re-thinking age criteria

**DOI:** 10.1186/1741-7015-12-83

**Published:** 2014-05-21

**Authors:** Barbara E Gibson, Gillian King, Shauna Kingsnorth, Patricia McKeever

**Affiliations:** 1 Department of Physical Therapy, University of Toronto, Toronto, Canada; 2 Bloorview Research Institute, Holland Bloorview Kids Rehabilitation Hospital, 160-500 University Avenue, Toronto, ON M5G 1 V7, Canada; 3 Occupational Science and Occupational Therapy, University of Toronto, 150 Kilgour Road, Toronto, ON M4G 1R8, Canada; 4 Bloomberg Faculty of Nursing, University of Toronto, 150 Kilgour Road, Toronto, ON M4G 1R8, Canada

**Keywords:** Complex care, Impairment, Long term care, Children and young people, Transitions, Life course, Disability, Chronic care, Home

## Abstract

**Background:**

Advances in lifesaving technologies and treatments make it possible for children with profound physical and cognitive impairments to survive into adulthood. Questions regarding how and where they should live are discussed rarely and, when they are, primarily focus on safety and/or containing costs. Since models of long-term care provision are age-based, children who reside in institutions are ‘discharged’ to adult facilities when they reach an arbitrary age. Such transfers may not be in the best interests of these young people or their families. Our aim in this debate is to highlight why age is a problematic criterion for placement decisions, with the goal of stimulating further research and inquiry.

**Discussion:**

Transfers from pediatric to adult institutions are driven primarily by funding arrangements and underpinned by stage-based theories of human development. Arguments supporting such transfers point to the value of communal living with same age peers, and engagement in age-appropriate activities. These goals are questionable for individuals who are minimally interactive and/or where equally worthy interactions are feasible in intergenerational settings. Instead their accommodation needs might more closely align with palliative care principles of supporting individuals and families to enjoy what they bring to each other’s lives and minimize suffering. Innovative models of ‘vertical care’ and ‘lifetime homes’, which enable continuous flexible services across the lifespan, are discussed as examples of alternative approaches requiring further debate and research.

**Summary:**

Entrenched funding and service models that require the transfer of profoundly impaired young people from pediatric to adult facilities need to be re-examined with considerations of best interests, needs, and preferences of individuals and their families. Questions of what constitutes a ‘good life’ for these individuals are tenacious and require further thought and research. Nevertheless, they need to be regarded as citizens of our human community deserving of a good life in whatever form that may take, in settings that enable them to flourish.

## Background

Although Western industrialized countries are focused on the health care implications of the growing numbers of older adults, there is another rising population with burgeoning health care needs – young people who have survived formerly fatal anomalies, injuries, or diseases [1-3]. Advances in medical technologies and lifesaving treatments make it possible for neonates, children and young adults with profound physical and cognitive impairments to survive into adulthood, but they rely on technologies, professionals and family caregivers for ongoing survival [4]. These children now account for almost a third of all child health spending in Canada and elsewhere [5], and their numbers are rising exponentially [6]. Providing care for this group is very expensive. In 2006, Noyes and colleagues determined that the average annual cost of maintaining one ventilator-dependent child in a British institution was £301,888 [7]. At the same time, they note that over the past 20 years much health policy has been oriented to containing escalating costs.

Persons with profound impairments who have survived previously fatal childhood conditions thus constitute another ‘aging population’. However, this population has received relatively little attention from policy makers and health researchers. Their ongoing survival and growing numbers are raising pressing questions regarding where and how they should live, and society’s obligations to this vulnerable group. Lives are saved through impressive technological advances but little attention is given to determining how best to support them throughout increasingly longer life spans. When quality of life issues are raised, the debate is focused almost exclusively on the ethics of using particular life saving measures (see, for example, the recent Supreme Court of Canada case, Cuthbertson v. Rasouli [8]). What is largely ignored is the everyday wellbeing of persons whose lives have been saved, and particularly those who live in institutional settings. Questions regarding how and where they should live are rarely discussed, and when they are, the primary focus is on safety and/or containing costs.

In this paper we focus on individuals who appear to have little understanding of verbal language, have ongoing high care needs, and have no capacity for self-support. The severely compromised physical repertoire of these individuals renders them minimally interactive. They require twenty-four hour care that includes combinations of assistance with bodily maintenance, reliance on life sustaining technologies, and/or skilled professional care. Diagnostic groups include, for example, severe cerebral palsy, traumatic brain injury, or brainstem stroke which have rendered individuals unable to move, gesture or speak; and individuals who are ‘locked in’, ‘minimally conscious’ or in a ‘persistent vegetative state’. In what follows we refer to these individuals collectively as ‘profoundly impaired’.

Current models of long-term care provision are age-based such that children receiving long-term institutional care are inevitably ‘discharged’ from pediatric institutions and moved to adult facilities when they reach adulthood. This move, which has been likened to an eviction, can be disruptive and upsetting for all involved – parents, young people and institutional care providers alike. We believe that such transfers neither reflect the best interests of young people nor family-centered care principles. Rather they seem driven primarily by funding arrangements that disallow adults to reside in facilities designated for the care of children (and vice versa), and are underpinned by traditional stage-based theories of human development [9]. Our aim in this debate paper is to initiate a discussion of whose needs are served or hindered when profoundly impaired people are grouped into age categories, and to question how placement^a^ decisions are made. Rather than provide definitive answers to these complex questions, we modestly aim to signpost some key parameters for discussion and further inquiry, and stimulate ethical reflection on how to accommodate (in the many senses of this word) these vulnerable persons.

## Discussion

### Age as a problematic criterion

In the last decade, the rhetoric of ‘transition’ has been used to frame discussions pertaining to transferring young people from pediatric to adult services and settings. Traditional transition initiatives typically focused on health services; however, they are increasingly concerned with ‘life transitions’ and support services enabling young people to take up adult social roles, thereby moving from one life stage (childhood) to another (adulthood) [10]. Conceptually, ‘transitions’ reflects a short-term view of life changes, whereas the term ‘trajectories’ provides a longer view of changes over the life span [11]. However, profoundly impaired children do not fit easily into these dominant formulations. Neither transitions nor trajectories are applicable or relevant to them in any traditional sense of increasing independence and changing roles commonly associated with adulthood.

We concur with Priestley [12] that adulthood is an identity category, a socially constructed division from childhood that is aligned with chronological age. As children age they are expected to acquire the abilities, privileges, responsibilities and characteristics associated with the prevailing socio-cultural understandings of adulthood. In most Western countries, adulthood is marked by achievement of residential and financial independence from parents, as well as emotional self-reliance, cognitive self-sufficiency and behavioral self-control [12,13]. Expectations of this ‘developmental progression’ from child to adult status are embedded in social norms, rituals and laws as well as educational, psychological and biomedical discourses [9].

There is little research or scholarship examining institutional placement and transfer for profoundly impaired young people who cannot assume adult social roles [14,15], with the exception of a growing body of work oriented towards moving children and young people out of institutions into the community [16-22]. Empirical research and policy reform point to the inadequacy of adult-oriented long-term care homes for younger adults, and have grounded calls for increased services to allow children to live in family homes and/or family-friendly group home settings [15,21,23,24].

Research has aimed to address the wellbeing of affected young people and their families, but in most cases has not directly considered the needs of the most profoundly impaired young people. Moreover, we have found no research questioning or investigating age-based criteria for institutional placement or transfer for this group. Across the literature there is a prevalent assumption that disabled youth share similar transition needs vis-à-vis transferring to adult services. For example, Doug *et al*. [25] conducted a systematic review of transitions services for young people with life limiting conditions and concluded that services needed to be ‘appropriate for chronological age and developmental stage’ (p.9). None of the 92 papers from seven countries in this systematic review appear to have raised the possibility that age criteria may not be applicable to all children and young people. The ‘transitions’ described included models where young people remained with a pediatric provider team, but invariably these models were construed as problematic and related to lack of available adult programs, and/or families and providers having trouble ‘letting go’ due to ‘emotional attachments’. Although we do not dispute that moving from pediatric to adult-based care may be beneficial for many young people, we argue that the universal application of age criteria is problematic. Profoundly disabled young people may have substantially different care needs that require individualized consideration.

Critiques of the use of age criteria and their role in structuring health and social services have come primarily from the social sciences [12,26-28] and the growing field of antidevelopmental psychology [9,29-34]. These critics assert that the stages of the life course (childhood, adulthood, old age) are culturally produced and grounded in assumptions derived from historical roots and misrecognized as biological facts [35]. These ideas have global resonance^b^ but closely align with Western notions of individualism and progress [32]. Meyer [36], for example, suggests that American social ‘problems’ might not be construed as problematic in societies less focused on the individual and an assumed proper life course. She notes that it is not surprising that systems and organizations perpetuate life stage divisions because they are designed to do so. Similarly, Priestly [12,35] has suggested that an ‘idealised’ life course trajectory that pivots around the notion of independent adulthood has defined the boundaries of welfare entitlements in the UK and elsewhere. He notes that age and disability categories have been important historical factors in the control of labor supply and continue to be used to determine health and welfare policies (for examples of age-based policies see [37-41]). Priestly [35] suggests that age-based transition policies have had the effect of relegating some people with disabilities to a liminal ‘nether world’ of unresolved transitions putatively designed to approximate adult roles.

The lives of profoundly impaired individuals do not conform to a developmental trajectory of progressive self-sufficiency from childhood to adulthood. Their impairments render it impossible to live or work independently, and they require ongoing intense services and supports throughout their lives. Moreover, except for physical/biological changes, a developmental perspective to human growth and adaptation over the lifespan has minimal relevance. The development of self-regulative capacity to adapt to different conditions and contexts [42] has little application to persons with complex, profound impairments.

It follows that moving profoundly impaired young people from one institutional setting to another solely on the basis of age may be ill-advised and even harmful to them and others. Such moves may needlessly disrupt their lives and those of their families [43] and sever established relationships with care providers that have developed over months or years [44]. Arguments supporting such transfers point to the value of communal living with same age peers, and engaging in age-appropriate activities. However, these worthy goals may be inappropriate for individuals who are minimally interactive and/or where positive interactions would be equally feasible in intergenerational settings. Age is only one possible characteristic shared with other residents and may be of little importance. Because long-term care settings are important interactive spaces for families, it may be as, or more, relevant to consider the similitude of family members’ needs and situations. Thus, ‘age’ and/or life stage are difficult criteria to use in support of institutional accommodation and care requirements.

### The importance of home

Because most profoundly impaired persons continuously (or episodically) live in institutional settings, moving from one setting to another may be inimical to their wellbeing. These facilities are ‘homes’ and comprise stable venues for ongoing family engagement. Long term relationships also develop with staff members who know the person’s needs and responses, have developed effective routines and uses of familiar devices, equipment and adaptations, and may be best positioned to ‘read’ non-verbal cues as rhythms of daily life become established over time [44,45]. Institutional homes, like any homes, are relational spaces composed of much more than bricks and mortar. They include the dynamics of the persons and processes that occur within them in the context of their local and larger communities. One’s home is fundamental in shaping everyday life by providing a sense of continuity, security and safety [15-18]. Ideally, homes are places where inhabitants establish and maintain relationships and trust. Homes have been conceptualized as ‘a space of comfort’ created in a never-ending process [46]. They can be places of healthcare work or familial relationships, places of comfort or, conversely, sites of abuse, pain and neglect. They are thus more than material structures because they reflect the presence, habits and effects of their occupants [47].

Given the significance of home to human wellbeing, questions of placement, discharge or transfer of profoundly impaired people from one institutional setting to another are clearly important. Changing homes is not just a change in locale, and involves more than procuring a bed space or providing routine health and care services. Housing is central to the health and wellbeing of all individuals and their families [48]. Homes are the locus of personal relationships which, in this instance, include care relationships [48]. This raises the question, ‘For whom is it a problem if profoundly impaired persons live in one long-term care setting throughout their lives?’ Certainly institutional lifetime homes are problematic in jurisdictions where prevailing institutional arrangements and public funding models separate child from adult care. Are there better ways and environments for these individuals and their families to live and flourish?

### Future directions

If age is not taken for granted as the cardinal criterion for placement decisions, other alternatives can be considered. As noted above, very little research has investigated the institutional care needs of profoundly impaired children and young people. Thus, rather than provide definitive solutions, in this section we signpost some possible considerations and emerging models that could ground further inquiry.

In previous work we have suggested that, at a minimum, all homes should promote individual dignity beyond the provision of safety and basic care [23]. Furthermore, Asch and colleagues [49] posit that institutional homes should be designed to enable continuity of services across the lifespan as residents’ care needs change over time. They also suggest that the typical ‘horizontal delivery’ of services, where all persons receive the same services in the same setting, be replaced with ‘vertical delivery’ facilities to meet residents’ needs as they age, with reasonably consistent cohorts of service providers.

Support for vertical care is further reflected in notions of lifetime homes for disabled people [50]. Lifetime homes are designed as adaptable spaces to accommodate individuals as they age and their functional abilities decline (or improve) [51]. Vertical care and lifetime homes are both consistent with the goal of continuous care over the life course. Currently, ‘life course’ is typically articulated as the period from early adulthood to old age, but we agree with Imrie [50] that this limited view should be expanded to include childhood. Regardless of age of onset, the idea that individuals with lifelong profound impairments could potentially benefit from living in stable environments warrants further investigation. Such environments may provide opportunities for the collective ‘home making’ activities of impaired individuals, families and care providers over time [52]. Research and pilot programs are needed to investigate these possible benefits and to inform policy and practice.

Decisions regarding where and how profoundly impaired individuals should live inevitably rest on questions of what constitutes a ‘good life’ for those unable to articulate their preferences. Recent neuro-imaging research has shown it is possible to communicate with some ‘behaviorally non-responsive’ individuals who have the ability to respond to commands through wilfully modulating their brain activity [53]. Such research raises questions about our limited understandings of the inner worlds of profoundly impaired individuals and their potential awareness of their surroundings. Their abilities to experience suffering, security or contentment may vary widely but cannot yet be easily discerned. In many cases, parents and caregivers learn to ‘read’ profoundly impaired young people over time, to understand when they are uncomfortable or in pain, or are experiencing pleasure or comfort [45]. Nevertheless, it is difficult to ascertain what gives meaning, contentment or joy to their lives, or if indeed these are the right questions to ask. As a starting point, we suggest that care should be oriented towards preventing or removing harms – pain, distress, insecurity and suffering - as much as it is possible to discern signs of these experiences. As part of this approach, decisions related to home placement or transfer need to consider carefully the potential harms associated with such moves.

Parents’ priorities provide some direction in establishing placement/transfer criteria as alternatives to age. Research conducted by Rabiee *et al*. [54] found that many parents of children with complex health care needs prioritized comfort and relief of pain over other outcomes, and saw comfort as necessary for achieving other outcomes, such as learning to eat by mouth, or increasing interactions. Children’s quality of life, even if parents’ could not easily define it, was important and prioritized over longevity. Similar research has demonstrated the importance of lasting, trusting partnerships between parents of disabled children and professionals [55]. Identified features of high quality partnerships included staff members who appeared to understand children’s conditions, skilfully met their needs, and treated them with respect and partnerships characterized by continuity of professional staff members [43,44,55].

To conclude, we suggest that decisions regarding placement for profoundly impaired persons should not be made using the limited criteria of age, physical safety and the provision of bodily care. The latter two provisions are necessary but insufficient conditions that reduce persons to objects of care and fail to address their inherent dignity *qua* human beings [56]. Second, ‘transitions’ models oriented to preparing children for the ‘next stage’ in life may be less appropriate for profoundly impaired young people who are not expected to change roles or activities in any traditional way. Instead, their accommodation needs might more closely align with palliative care goals to support individuals and families to ‘live well in the present,’ to enjoy what they bring to each other’s lives, support meaningful interactions and minimize suffering and family burdens [57]. We suggest that, in accord with the World Health Organization’s principles [58], care for profoundly impaired individuals needs to be provided across the life course, be focused on relieving human distress, support individuals and their families, and be directed towards body, mind and spirit. Within the context of these principles, arbitrary age criteria for transferring vulnerable young people to adult facilities are, at best, insufficient. Transfer policies should be re-examined in terms of how homes enable or impede the care of profoundly impaired individuals and support family-centered care.

For the foreseeable future, at least some profoundly impaired young people will continue to require institution-based care. Given this reality, there is a pressing need for interdisciplinary research to develop and evaluate alternative service models. We envision partnerships between child health researchers, social scientists and policy makers working closely with care providers and families to determine what best meets their needs. A useful starting place could be targeted pilot case study research with families who wish to remain in pediatric facilities, working in partnership to develop models of care delivery and family supports.

## Summary

In this debates paper, we have suggested that age is an arbitrary and inappropriate criterion for discharging and displacing profoundly impaired individuals from pediatric to adult institutional long-term care settings. Entrenched funding and service models that require such dis-placements need to be re-examined with considerations of best interests, needs and preferences of individuals and families. Innovative models such as vertical care and life time homes merit serious deliberation and further research as increasing numbers of children are surviving previously fatal conditions. Questions of what constitutes a ‘good life’ for these individuals are tenacious and require further thought and innovative research. Nevertheless, profoundly impaired persons must be regarded in terms other than ‘bed blockers’ or ‘tragedies’. They are citizens of our human community deserving of a good life in whatever forms that may take. To that end we welcome further dialogue and debate.

## Endnotes

^a^ By ‘placement’ we are referring to the common rhetoric of health professionals charged with finding appropriate living and care arrangements for people who need health and/or attendant care services and, for whatever reason, need to leave their current arrangements.

^b^ See for example the World Health Organization Life Course Model used to train health and social care professionals [59]. The model divides the life course into three stages (Early/Adult/Older) and describes a trajectory of functional capacities that peak in adulthood followed by a decline. Disability is associated with aging, and interventions are positioned as a means to slow or reverse decline. The life trajectories of profoundly impaired young people bear little resemblance to this assumed life course.

## Competing interests

The authors declare that they have no competing interests.

## Authors’ contributions

BG drafted the manuscript. PMcK conceived of the paper. All authors contributed to the development of the ideas and to the writing. All authors read and approved the final manuscript.

## Authors’ information

BG is an Associate Professor in the Department of Physical Therapy, University of Toronto, and a Senior Scientist at the Bloorview Research Institute at the Holland Bloorview Kids Rehabilitation Hospital in Toronto, Canada, where she holds the Bloorview Children’s Hospital Foundation Chair in Childhood Disability Studies. Her research examines the social and ethical dimensions of childhood disability and rehabilitation, particularly the norms embedded in rehabilitation practices and the effects on disabled children and young people. GK is a Senior Scientist at the Bloorview Research Institute. She has a PhD in Social Psychology and conducts research on psychosocial issues of children with disabilities and their families. She has published articles on resiliency, quality of life, meaning in life, family-centered service, youth transitions, and child and family wellbeing and adaptation. SK is a Clinical Study Investigator at the Bloorview Research Institute and Lead of Evidence to Care at the Teaching & Learning Institute at the Holland Bloorview Kids Rehabilitation Hospital in Toronto, Canada. Building on her background in Developmental Psychology, her research interests focus on enhancing the participation and inclusion of youth with physical disabilities and/or medical complexity, including their transition to adulthood, impact of alternative and complementary therapies and service models supporting continuity of care. PMcK is a Senior Scientist, Bloorview Research Institute, Holland Bloorview Kids Rehabilitation Hospital, and Professor, Lawrence S. Bloomberg Faculty of Nursing, University of Toronto. Her interdisciplinary program of research focuses on disabled children, their sense of embodiment, the technologies they use, their care providers and the places where they live, attend school, and/or receive care. Her research addresses the physical, social and policy barriers to inclusion that disabled children and their families encounter.
